# Neuropsychiatric symptoms and altered sleep quality in cerebral small vessel disease

**DOI:** 10.3389/fpsyt.2022.882922

**Published:** 2022-08-16

**Authors:** Xi Li, Rong-Rong Qin, Jian Chen, Hai-Fei Jiang, Pan Tang, Yu-Jing Wang, Dong-Wu Xu, Tao Xu, Ti-Fei Yuan

**Affiliations:** ^1^Department of Neurology, Affiliated Tongzhou Hospital of Nantong University, Nantong, China; ^2^School of Mental Health, Wenzhou Medical University, Wenzhou, China; ^3^Department of Anesthesiology, Affiliated Shanghai Sixth People’s Hospital, Shanghai Jiao Tong University, Shanghai, China; ^4^Shanghai Key Laboratory of Psychotic Disorders, Shanghai Mental Health Center, Shanghai Jiao Tong University School of Medicine, Shanghai, China; ^5^Co-innovation Center of Neuroregeneration, Nantong University, Nantong, China; ^6^Shanghai Key Laboratory of Anesthesiology and Brain Functional Modulation, Translational Research Institute of Brain and Brain-Like Intelligence, Shanghai Fourth People’s Hospital Affiliated to Tongji University School of Medicine, Shanghai, China

**Keywords:** cerebral small vessel disease, aging, neuropsychiatric symptoms, depression, sleep disturbances

## Abstract

**Background:**

Sleep disturbance and neuropsychiatric symptoms are common clinical symptoms of cerebral small vessel disease (CSVD), but the underlying mechanism is unclear. Here, we investigated the relationship between sleep quality and neuropsychiatric performance in patients with CSVD.

**Methods:**

A total of 30 patients with CSVD and 35 healthy controls (HCs) were recruited. The 13-item Beck Depression Inventory (BDI-13), Beck Anxiety Inventory (BAI), and Symptom Check List 90 (SCL90) were used to assess depression, anxiety, and other psychological symptoms, respectively. Sleep quality was assessed using Pittsburgh Sleep Quality Index (PSQI), and cognitive function was tested using Montreal Cognitive Assessment (MoCA).

**Results:**

When compared to the HC group, the patients with CSVD showed increased anxiety and neuropsychiatric symptoms, worse sleep quality, and impaired cognition (*p* < 0.05). The prevalence of comorbid poor sleep quality in the patients with CSVD was approximately 46%. The patients with CSVD with poor sleep quality also had more severe neuropsychiatric symptoms. After controlling for demographic variables, sex and anxiety significantly predicted sleep quality.

**Conclusion:**

This study suggests that the prevalence of CSVD with poor sleep quality is high, and that sex and anxiety are independent risk factors for CSVD comorbid sleep quality.

## Introduction

Cerebral small vessel disease (CSVD) is a common cerebrovascular disease and refers to a series of clinical, imaging, and pathological syndromes caused by various causes affecting small arteries, arterioles, capillaries, venules, and small veins in the brain (1). Due to complex clinical symptoms and the insidious and slow onset of the disease, it is likely to cause confusion in diagnosis and treatment, which may lead to continued deterioration of the disease (2). The past mainly focused on cognitive function in CSVD, including early impairment of attention, processing speed and executive function, as well as the relative integrity of memory function (3, 4). Non-cognitive psychiatric symptoms are often ignored. In fact, comorbidities of sleep disturbance and cerebrovascular diseases are very common (5).

Poor sleep quality is considered to be one of the non-traditional risk factors for cardiovascular and cerebrovascular diseases (6, 7), and it is not only associated with cognitive decline (8) but also with development or exacerbation of other psychiatric symptoms (9, 10). Prosperous sleep quality plays a vital role in consolidating memory, regulating immunity, and balancing homeostasis (11). Therefore, early identification of sleep disturbance and clinical intervention are of great significance to alleviate the speed of cognitive impairment, improve the mental and psychiatric symptoms of patients, and reduce the burden on caregivers. In addition, neuropsychiatric symptoms are major factors causing a decline in quality of life and burden on caregivers of patients with CSVD. Previous studies suggested sleep disturbance was closely related to neuropsychiatric symptoms in Alzheimer’s disease (12). Improving sleep quality and preventing neuropsychiatric symptoms are an important part of the clinical management of aging patients. However, little is known about the relationship between sleep quality and neuropsychiatric symptoms in patients with CSVD.

In this study, we comprehensively described common neuropsychiatric symptoms, sleep quality, and cognitive performance in patients with CSVD, and further investigated the relationship between sleep quality and neuropsychiatric symptoms in patients with CSVD. We hypothesized that (1) patients with CSVD would have more severe neuropsychiatric symptoms than healthy controls (HCs) and (2) sleep quality in patients with CSVD would be related to neuropsychiatric characteristics.

## Material and methods

### Participants

Thirty patients diagnosed with CSVD were recruited in the Department of Neurology, Nantong People’s Hospital, China from August 2020 to December 2020. Inclusion criteria included age ≥18 years, presence of CSVD (microbleeds, perivascular spaces, and recent small subcortical infarcts) on magnetic resonance imaging (MRI), communication skills, and informed consent. Exclusion criteria included (1) contraindication to MRI, (2) severe renal or hepatic disease, cancer, or critical illness, (3) neurological disorders such as epilepsy or large vessel disease, (4) history of diagnosed Alzheimer’s disease and dementia, (5) history of psychiatry disorders and alcohol or substance dependence, and (6) substantial communication deficits.

Thirty-five healthy participants from the support service department in the same hospital were recruited as HCs. Demographic variables were matched with patients in terms of gender, age, and education years. The inclusion criteria for HCs were (1) age ≥18 years, (2) ability to communicate, (3) no history of major diseases and psychiatric or psychoactive substance use and no serious brain injury or implant, (4) no history of stroke or dementia, and (5) willingness to provide informed consent.

### Neuropsychiatric evaluation

All the participants underwent neuropsychological evaluations by two experienced psychiatrists. Depression and anxiety symptoms were measured with the 13-item Beck Depression Inventory (BDI-13) (13), and the Beck Anxiety Inventory (BAI) (14). Both the BDI-13 and the BAI are four-point Likert scales; the former has 13 items and the latter consists of 21 items. High-scale scores indicate more severe depressive and anxiety symptoms. The Symptom Check List 90 (SCL90) is a widely used scale for assessing psychiatric symptoms from multiple perspectives (15). We used six subscales: somatization, obsession-compulsion, interpersonal sensitivity, hostility, phobic anxiety, and paranoid ideation. It was rated on five-point scales (1–5), and a high score meant high severity of mental symptoms.

### Cognitive function

Two trained doctors used Montreal Cognitive Assessment (MoCA) to assess the cognitive function of every participant. MoCA is a highly sensitive assessment tool for rapid screening of mild cognitive dysfunction (16). MoCA contains seven subdimensions: visuospatial, naming, attention, language, abstraction, recall, and orientation. The sum of all item scores is the total score. For those with no more than 12 years of education, one point is added. The maximum score is 30 points.

### Sleep quality

Pittsburgh Sleep Quality Index (PSQI) was used to evaluate the sleep quality of the participants in the past month. It comprised 19 items in 7 parts, namely, subjective sleep quality, sleep latency, sleep duration, habitual sleep efficiency, sleep disturbances, use of sleeping medication, and daytime dysfunction (17). Each dimension was graded from 0 to 3 points, with higher sum scores indicating worse sleep quality. Patients with PSQI scores greater than 7 were considered to have poor sleep quality (18).

### Statistical analysis

Normality tests were conducted for all continuous variables. For comparison of demographics and clinical information between the groups, Chi-square tests for categorical variables and independent-samples *t*-tests or Mann–Whitney U tests for continuous variables were performed. Cohen’s *d* (for *t*-tests) and Freeman’s theta (for Mann–Whitney U tests) were calculated separately to represent the effect size. A Spearman correlation analysis was conducted to explore the association between PSQI and neuropsychiatric symptoms. Furthermore, a linear regression analysis was performed to identify significant predictive variables associated with PSQI. The data were expressed as mean + standard deviation (SD). All the statistical analyses were performed using SPSS 21.0. All statistical tests were two-tailed and *p* < 0.05 was considered statistically significant.

## Results

### Demographic information and psychiatric symptoms of patients with cerebral small vessel disease and healthy controls

Table 1 shows no significant differences in sex, age, and education years between the patients with CSVD and the HCs (all *p* > 0.05). The patients with CSVD had higher scores on the BDI-13 (*z* = −2.059, *p* < 0.05, Freeman’s theta = 0.28), BAI (*z* = −3.632, *p* < 0.001, Freeman’s theta = 0.521), and PSQI (*t* = 3.735, *p* < 0.001, Cohen’s *d* = 0.941) than the HCs. Moreover, there are significant differences in SCL90 including total score (*z* = −3.156, *p* < 0.01, Freeman’s theta = 0.456), somatization (*z* = −4.383, *p* < 0.001, Freeman’s theta = 0.628), and obsessive-compulsive (*z* = −2.206, *p* < 0.05, Freeman’s theta = 0.316) between the patients with CSVD and the HCs. The other subdimensions of SCL90 did not differ significantly. In addition, the patients with CSVD performed worse on MoCA than the HCs (*t* = −2.866, *p* < 0.01, Cohen’s *d* = 0.96).

**TABLE 1:** Demographic information and psychiatric symptoms of the patients with CSVD and healthy controls.

	CSVD	HC	χ ^2^*/t/z*	*p*	Cohen’s *d/*Freeman’s theta
	
	(*N* = 30)	(*N* = 35)			
Sex (male, female)	20, 10	15, 20	3.685	0.055	
Age	64.63 (7.582)	61.49 (4.829)	1.959	0.056	
Education (years)	7.18 (4.014)	6.27 (3.497)	0.979	0.331	0.242
BDI-13	2.6 (3.944)	1.03 (1.706)	–2.059	*p* < 0.05	0.280
BAI	27.17 (6.097)	23.17 (2.629)	–3.632	*p* < 0.001	0.521
PSQI	8.2 (4.0292)	4.914 (2.853)	3.735	*p* < 0.001	0.941
MoCA	19.8 (4.382)	22.49 (3.147)	–2.866	*p* < 0.01	0.960
SCL90	66.17 (11.108)	58.23 (7.655)	–3.156	*p* < 0.01	0.456
Somatization	17.97 (3.419)	14.31 (2.564)	–4.383	*p* < 0.001	0.628
Obsessive-compulsive	13.77 (2.674)	12.4 (2.464)	–2.206	*p* < 0.05	0.316
Interpersonal sensitivity	11.17 (3.03)	10.51 (2.02)	–0.512	0.609	0.071
Hostility	7.67 (2.139)	6.69 (0.932)	–1.649	0.099	0.221
Phobic anxiety	8.5 (2.933)	7.54 (1.314)	–1.561	0.118	0.178
Paranoid ideation	7.1 (1.845)	6.77 (1.114)	–0.139	0.89	0.017

Values are presented as mean ± SD. Age, education (years), MoCA, and PSQI conformed to normal distribution. BDI-13, BAI, SCL90, somatization, obsessive-compulsive, interpersonal sensitivity, hostility, phobic anxiety, and paranoid ideation were not normally distributed. CSVD, cerebral small vessel disease; HCs, healthy controls; BDI-13, 13-item Beck Depression Inventory; BAI, Beck Anxiety Inventory; PSQI, Pittsburgh Sleep Quality Index; MoCA, Chinese version 3.3 of the Montreal Cognitive Assessment; SCL90, Symptom Checklist 90.

### Demographic and neuropsychiatric performance of patients with and without poor sleep quality

Table 2 shows that 14 of the 30 patients had co-morbid poor sleep quality. Sex and age are matched between patients with and without poor sleep quality (all *p* > 0.05). The patients with poor sleep quality had lower education years (*z* = −2.188, *p* < 0.05, Cohen’s *d* = −0.801) and had higher scores on the BDI-13 (*z* = 2.441, *p* < 0.05, Freeman’s theta = 0.429) and BAI (*z* = 2.788, *p* < 0.05, Cohen’s *d* = 1.048), and SCL90 total score (*t* = 2.231, *p* < 0.05, Cohen’s *d* = 0.832). This means that the patients with CSVD with poor sleep have worse mood and neuropsychiatric symptoms.

**TABLE 2:** Demographic and neuropsychiatric performance of patients with and without poor sleep quality.

	With poor sleep	Without poor sleep	χ ^2^*/t/z*	*p*	Cohen’s *d*/Freeman’s theta
	
	(*N* = 14)	(*N* = 16)			
Sex (male, female)	7/7	13/3	3.281	0.07	
Age	65.5 (5.288)	63.88 (9.251)	0.6	0.554	0.215
Education (years)	5.57 (3.715)	8.59 (3.826)	–2.188	<0.05	-0.801
BDI-13	4.43 (5.155)	1 (1.095)	2.441	<0.05	0.429
BAI	30.29 (7.508)	24.44 (2.449)	2.788	<0.05	1.048
MoCA	19 (4.057)	20.5 (4.662)	–0.933	0.359	-0.343
SCL90	70.86 (13.26)	62.06 (6.904)	2.231	<0.05	0.832
Somatization	19.07 (3.362)	17 (3.266)	1.71	0.098	0.625
Obsessive-compulsive	14.07 (2.731)	13.5 (2.683)	0.577	0.568	0.211
Interpersonal sensitivity	12 (3.53)	10.44 (2.394)	1.435	0.258	0.232
Hostility	8.43 (2.652)	7 (1.317)	1.828	0.275	0.223
Phobic anxiety	9.71 (3.911)	7.44 (0.892)	2.13	0.083	0.321
Paranoid ideation	7.57 (2.243)	6.69 (1.352)	1.284	0.244	0.210

Values are presented as mean ± SD. Age, education (years), BAI, MoCA, SCL90, somatization, and obsessive-compulsive conformed to normal distribution. BDI-13, interpersonal sensitivity, hostility, phobic anxiety, and paranoid ideation were not normally distributed.

### Relationship between Pittsburgh Sleep Quality Index and neuropsychological performance in the cerebral small vessel disease group

**FIGURE 1:**
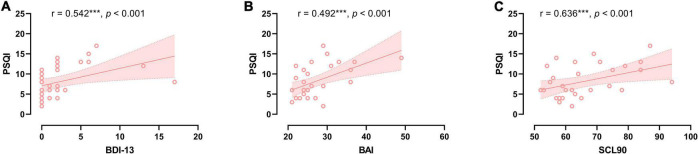
The relationship between sleep quality and neuropsychological severity in CSVD group. **(A)** Correlation between sleep quality and depression severity. **(B)** Correlation between sleep quality and anxiety severity. **(C)** Correlation between sleep quality and SCL90 score.

In the CSVD group, PSQI score was significantly positively associated with BDI-13 level (*r* = 0.542, *p* < 0.001), BAI level (*r* = 0.492, *p* < 0.001), and SCL90 total score (*r* = 0.636, *p* < 0.001) (refer to Figure 1). Further multiple regression with BDI-13, BAI, SCL90 as independent variables and PSQI as dependent variables showed that after controlling for sex, age, and education years, sex (β = 0.418, *p* = 0.015) and BAI scores (β = 0.428, *p* = 0.017) still significantly predicted PSQI scores in the patients with CSVD. The model was significant (*F* = 4.736, *p* = 0.003) and could explain 43.8% of the variance in PSQI scores (refer to Table 3). Sex and anxiety are independent risk factors for sleep quality in the patients with CSVD.

**TABLE 3:** Multivariate linear regression to predict sleep quality.

	Coefficients							95.0% confidence interval for *B*	
	*B*	SE	β	*t*	*p*	*F*	Adjusted *R*^2^	Lower bound	Upper bound
Sex	3.51	1.338	0.418	2.624	0.015	4.763**	0.438	0.743	6.278
Age	0.085	0.078	0.161	1.094	0.285			–0.076	0.247
Education(years)	–0.119	0.162	–0.119	–0.736	0.469			–0.454	0.216
BDI-13	0.305	0.212	0.298	1.436	0.165			–0.134	0.744
BAI	0.283	0.11	0.428	2.567	0.017			0.055	0.511
SCL90	–0.003	0.075	–0.009	–0.044	0.966			–0.158	0.152

**p < 0.01.

## Discussion

This study reported that the patients with CSVD in our sample had significantly higher depression and anxiety status than the HCs, which is consistent with many previous studies (19). Usually, CSVD may lead to mood disorders by disruption of prefrontal subcortical structures or modulation pathways related to emotion regulation (20). In patients with Alzheimer’s disease and vascular dementia, the volume of periventricular white matter hyperintensity (WMH) is related to depression and anxiety (21), and this correlation is stronger in the frontal (22). Moreover, other psychiatric symptoms were significantly more severe in patients with CSVD than those with HC, including SCL90 total scores, somatization and obsessive-compulsive, placing a serious burden on quality of life and health care usage (23). In addition, the patients with CSVD had significantly worse sleep quality and cognitive impairment generally. These findings support the statement that multiple neuropsychiatric symptoms may occur in the early stages of CSVD.

The prevalence of comorbid poor sleep quality in patients with CSVD was approximately 46%, significantly higher than the 35.9% prevalence of sleep disorders in older Chinese adults (24), and close to the 50% prevalence recently reported for patients with cerebrovascular disease (8). This may indicate that sleep disturbance is one of the core non-cognitive symptoms of CSVD. In a community elderly people sample, sleep quality is associated with WMH even after controlling for sociodemographic variables and cardiovascular risk factors (25). The disruption of the white matter structural connectivity network in patients with insomnia syndrome, suggests (26), suggesting that it may be an intermediate mechanism between insomnia and CSVD pathology. Moreover, patients with poor sleep quality also had more severe depression, anxiety, and other mental symptoms. Due to the disease being chronic, long-term poor sleep quality or neuropsychiatric symptoms have become the key point of poor quality of life in patients with CSVD, and these symptoms should be the goal of treatment in the clinical setting.

Furthermore, the relationship of neuropsychiatric symptoms with worse sleep quality might suggest that neuropsychiatric symptoms are related to underlying CSVD burden. Numerous studies have reported that mood and neuropsychiatric symptoms are closely associated with sleep quality (27). Our current results suggested that depression, anxiety, and neuropsychiatric scores were significantly increased in patients with CSVD with poor sleep quality. Furthermore, the regression analysis showed that after controlling for sex, age, and education years, sex and BAI scores were still significantly predicting sleep quality. Female patients are susceptible to CSVD comorbid sleep disturbances, and anxiety level can also positively affect the quality of sleep of patients. Previous studies have also found that the prevalence of sleep disorders was significantly higher in women than in men (28), probably attributed to changes in female hormone levels (29) and effects of perceived stress in household chores. These findings remind us that in addition to relieving physical discomfort, reducing the occurrence or severity of neuropsychiatric impairment may be another key goal of clinical treatment. Every effective intervention that can improve the neuropsychological status of patients with CSVD will help reduce the burden on individuals and society, and it would be a potential step to reduce the incidence of comorbid sleep disturbance in CSVD.

Nevertheless, several limitations in this study should be taken into consideration. First, our sample size was relatively small, and the prevalence of CSVD combined with poor sleep should be generalized carefully. For verification, subsequent studies need to further expand the sample size. Second, there are many subcategories of poor sleep, and we only consider the sleep quality of patients generally. Future sleep research on CSVD may consider accurate classification and assessment. Third, the current cross-sectional study neither described the dynamic psychiatric symptoms during the entire progression of the disease nor showed the causal interrelationship between neuropsychiatric symptoms and sleep quality. In future studies, conducting a longitudinal study for disease changes and progress of poor sleep quality is undoubtedly interesting and important.

In conclusion, the study demonstrated that patients with poor sleep quality in CSVD group had more serious neuropsychological symptoms. Sex and anxiety could significantly predict sleep quality. With the aging population and influence of sociopsychological factors, poor sleep quality and CSVD will become a heavy social burden. Focusing on the early sleep quality of patients with CSVD is beneficial to the overall assessment, prognosis judgment, and intervention management of the disease, delaying the progression of cognitive dysfunction, reducing the risk of stroke and dementia, and improving the quality of life of patients.

## Data availability statement

The raw data supporting the conclusions of this article will be made available by the authors, without undue reservation.

## Ethics statement

Ethical review and approval was not required for the study on human participants in accordance with the local legislation and institutional requirements. The patients/participants provided their written informed consent to participate in this study.

## Author contributions

T-FY, TX, and D-WX designed the study. XL, PT, and Y-JW collected the data. JC and H-FJ interpreted the results. XL and R-RQ searched the literature and analyzed the data. All authors contributed to the writing and revisions.
